# Mesenteric Fibromatosis Presenting as a Diagnostic Dilemma: A Rare Differential Diagnosis of Right Iliac Fossa Mass in an Eleven Year Old—A Rare Case Report

**DOI:** 10.1155/2013/569578

**Published:** 2013-12-02

**Authors:** Abhinav Mahajan, Mohinder Singh, Anoop Varma, Gunjeet Singh Sandhu, Malwinder Singh, Rupesh Nagori

**Affiliations:** Department of Surgery, Government Medical College and Rajindra Hospital, Patiala, Punjab 147001, India

## Abstract

An eleven-year-old boy presented with a mass in the right iliac fossa for the last 21 days associated with pain, fever, anorexia, and nausea. The patient was thoroughly investigated and contrast-enhanced CT abdomen revealed a well-defined mass in the region of right iliac fossa. Exploratory laparotomy was done and a mass measuring 10 cm in diameter arising from mesentery of proximal ileum and adherent with the wall of ileum was seen. Resection and anastomosis were done. Histopathological examination showed mesenteric fibromatosis. Postoperatively, patient was well and 3-month followup showed normal recovery.

## 1. Introduction

Mesenteric fibromatosis is a part of the clinical-pathologic spectrum of deep fibromatoses. The deep fibromatoses encompass a group of benign fibroproliferative processes that are locally aggressive and have the capacity to infiltrate or recur but not metastasize. The deep fibromatoses are classified by anatomic location because they may arise from intraabdominal sites (mesenteric, pelvic, and retroperitoneal fibromatosis), the deep soft tissues of the abdominal wall (abdominal fibromatosis), and deep within extraabdominal soft tissues (extraabdominal fibromatosis) [1]. The small bowel mesentery is the most common site of origin of intraabdominal fibromatosis. Consequently, the terms mesenteric fibromatosis or mesenteric desmoid tumor are most often applied to this entity [2]. 

Mesenteric fibromatosis occurs in a wide age range of patients, 14–75 years of age (mean, 41 years), and has no gender or race predilection [2]. In contrast, abdominal fibromatosis occurs most commonly in young women, 20–30 years of age [3]. Most cases of mesenteric fibromatosis manifest sporadically. Thirteen percent of patients with mesenteric fibromatosis have familial adenomatous polyposis (FAP), specifically, the Gardner syndrome variant of FAP [2, 4].

 Prior abdominal surgery is an important risk factor for the development of mesenteric fibromatosis in patients with FAP. Eighty-three percent of patients with FAP and mesenteric fibromatosis have a history of abdominal surgery, most commonly a total colectomy [4].

The presenting clinical signs and symptoms of mesenteric fibromatosis are often related to the small bowel. Patients may complain of abdominal pain or a palpable abdominal mass or come to clinical attention because of complications such as gastrointestinal bleeding, small bowel obstruction, fistula formation, or bowel perforation [2, 5].

## 2. Case Report

**Figure 1:**
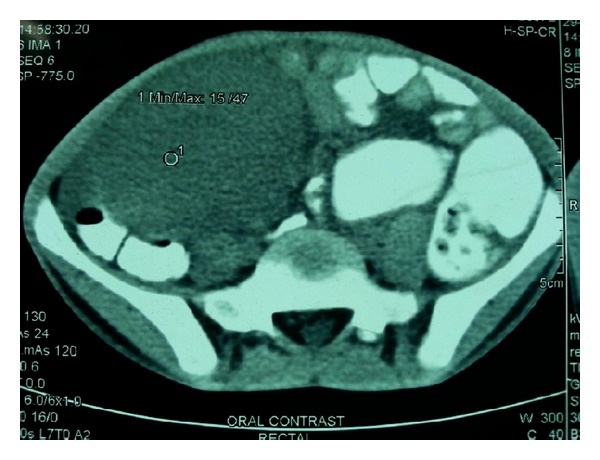
CT picture showing a well-defined mass in right iliac fossa.

A 11-year-old boy was admitted to paediatric ward with complaint of fever one month earlier. Fever was low grade and associated with decreased appetite. Then, patient noticed a mass in right iliac fossa 21 days back which was approximately 10 × 10 cm in size, mobile, firm, and globular associated with pain in right iliac fossa. He had no prior history of abdominal trauma or surgery. Patient was thoroughly investigated. All routine baseline investigations were found to be normal except that patient was anaemic (Hb = 7 g%); ESR was found to be raised at 140 mm in 1st hour by Westergren method. Contrast-enhanced CT abdomen was done which showed well-defined soft tissue mass in the peritoneal cavity in the region of right iliac fossa (Figure 1). No obvious abdominal lymphadenopathy was seen.

**Figure 2:**
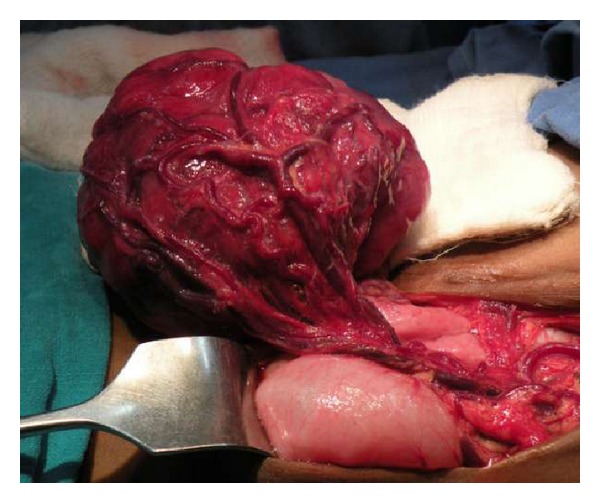
Lobulated mass arising from mesentery of proximal ileum.

**Figure 3:**
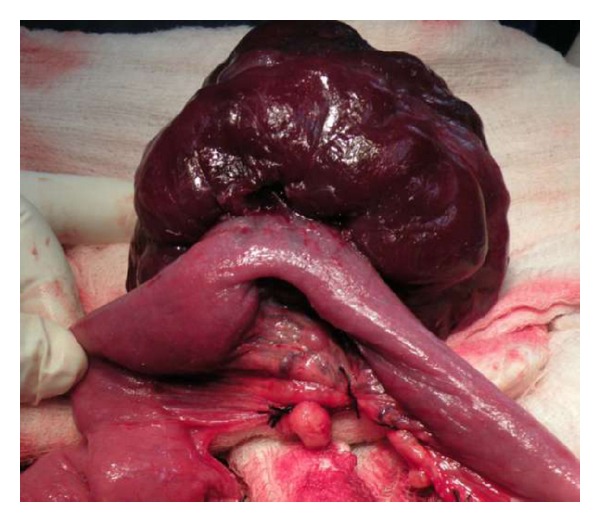
Well vascularised mass adherent to the bowel wall.

**Figure 4:**
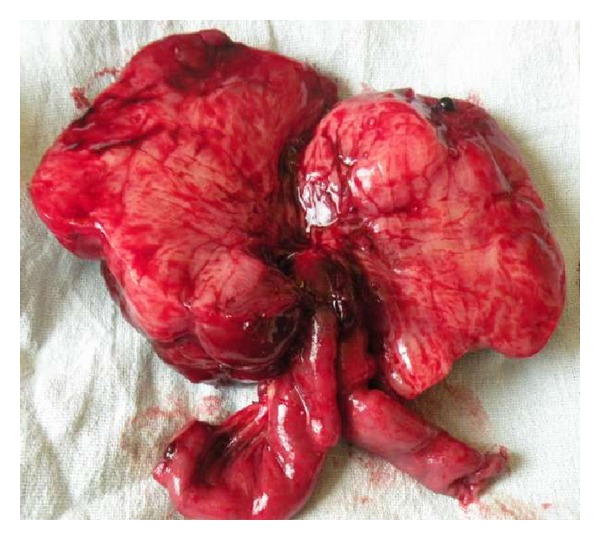
Cut section of the mass with the resected part of proximal ileum.

Patient was transfused with leucodepleted packed red cells and prepared for exploratory laparotomy under general anaesthesia. A well-vascularised mass measuring 10 cm in diameter was found arising from the mesentery of proximal ileum (Figure 2). It was adherent with the bowel wall as well (Figure 3). 10 cm of ileum was resected with the mass and anastomosis was done. The rest of the gut was normal. Mass was lobulated, and firm, and on cutting it had gritty sensation (Figure 4). Histopathological examination confirmed the diagnosis of aggressive fibromatosis. Postoperative period was uneventful and patient recovered well.

## 3. Discussion

Mass in the right iliac fossa has innumerable differential diagnosis ranging from mass arising from abdominal wall to peritoneal cavity to retroperitoneum. These include common disease processes like appendicular mass or abscess, ileocaecal tuberculosis, carcinoma caecum, intussusception, and lymphoma to some rare diagnosis like retroperitoneal sarcoma, iliac artery aneurysm, or chondrosarcoma of iliac crest. In females right iliac fossa mass may be due to ovarian cyst or tuboovarian mass. The present case discusses the rare possibility of mesenteric fibromatosis presenting as a right iliac fossa mass because it has never been mentioned in this differential diagnosis.

The majority of patients with mesenteric fibromatosis remain clinically asymptomatic, with little or no focal symptoms until later in their course, at which stage they complain of abdominal pain and discomfort, constipation, vomiting, and organ compression symptoms, such as small bowel obstruction and hydronephrosis [6]. As there is no classical symptomatology related to mesenteric fibromatosis, the diagnosis is confirmed only after the histological analysis of the tumor. Imaging remains the mainstay of preoperative investigations to establish a working diagnosis of mesenteric fibromatosis. The sonographic features of mesenteric fibromatosis are nonspecific and chiefly dependent on collagen and fibroblast content and intralesional vascularity of the tumor [7]. Due to the tendency of aggressive mesenteric fibromatosis to invade adjacent structures, CT scan is considered the first-line imaging modality for identifying, characterizing, and staging fibromatosis. On CT, these tumors appear as a soft tissue mass displacing/involving surrounding viscera, usually appearing as encasement of bowel loops [8]. Although the mass may appear well circumscribed, it often has irregular margins reflecting its infiltrative nature. 

Microscopically, mesenteric fibromatosis is characterized by a spatially homogenous proliferation of wavy spindle cells without atypia, associated with collagen among dilated vessels. The mitotic count is relatively low with no evidence of necrosis and nuclear dedifferentiation [9]. 

Wide field surgical excision is the first-line treatment for most mesenteric fibromatosis [10]. As noted in our case, the majority of these lesions require resection of the attached segment of the bowel [11]. Radiotherapy may be used before surgery in cases of recurrence and inoperable lesions to shrink the tumor and make it operable. Adjuvant radiation therapy reduces recurrence of mesenteric fibromatosis to 20%–40%, compared to 40%–70% with resection only [12]. In cases where surgery and radiotherapy are not rewarded by the desired success, systemic therapy with pharmacological agents (antiproliferative and cytotoxic drugs) can be employed, including estrogen receptor antagonist tamoxifen, nonsteroidal anti-inflammatory drugs agent sulindac, and chemotherapy with dactinomycin, vincristine, and cyclophosphamide, singly or in combination, with varying success. Despite all these agents, surgical excision is the gold standard primary treatment for mesenteric fibromatosis.

To conclude, mesenteric fibromatoses may present with bizarre clinical features and demonstrate a wide spectrum of imaging and histological spectra. Treating physicians should have a high index of suspicion while managing a patient with an abdominal mass and consider this entity in the differential diagnosis of a right iliac fossa mass as well.
